# DC Subsets Regulate Humoral Immune Responses by Supporting the Differentiation of Distinct Tfh Cells

**DOI:** 10.3389/fimmu.2019.01134

**Published:** 2019-05-27

**Authors:** Aurélie Bouteau, Jérôme Kervevan, Qingtai Su, Sandra M. Zurawski, Vanessa Contreras, Nathalie Dereuddre-Bosquet, Roger Le Grand, Gerard Zurawski, Sylvain Cardinaud, Yves Levy, Botond Z. Igyártó

**Affiliations:** ^1^Baylor Scott & White Research Institute, Baylor Institute for Immunology Research, Dallas, TX, United States; ^2^Institute of Biomedical Studies, Baylor University, Waco, TX, United States; ^3^Vaccine Research Institute, Créteil, France; ^4^INSERM, Unité U955, Institut Mondor de Recherche Biomédicale, Créteil, France; ^5^Faculté de Médecine, Université Paris-Est Créteil, Créteil, France; ^6^CEA—Université Paris Sud 11—INSERM U1184, Immunology of Viral Infections and Autoimmune Diseases, IDMIT Department, IBFJ, Fontenay-aux-Roses & Le Kremlin-Bicêtre, France

**Keywords:** dendritic cells, humoral immune response, Tfh cell, B cell, regulation

## Abstract

To determine the contribution of skin DC subsets in the regulation of humoral immunity, we used a well-characterized antigen targeting system to limit antigen availability and presentation to certain skin-derived DC subsets. Here we show that delivery of foreign antigen to steady state Langerhans cells (LCs) and cDC1s through the same receptor (Langerin) led to, respectively, robust vs. minimal-to-null humoral immune response. LCs, unlike cDC1s, supported the formation of germinal center T follicular helper cells (GC-Tfh) antigen dose-dependently and then, likely licensed by these T cells, some of the LCs migrated to the B cell area to initiate B cell responses. Furthermore, we found that the cDC1s, probably through their superior T cell activation capacity, prevented the LCs from inducing GC-Tfh cells and humoral immune responses. We further show that targeted delivery of cytokines to DCs can be used to modulate DC-induced humoral immune responses, which has important therapeutic potential. Finally, we show that human LCs, unlike monocyte-derived DCs, can support GC Tfh generation in an *in vitro* autologous system; and in agreement with mouse data, we provide evidence in NHP studies that targeting LCs without adjuvants is an effective way to induce antibody responses, but does not trigger CD8^+^ T cell responses. Our findings suggest that the major limitations of some relatively ineffective vaccines currently in use or in development might be that (1) they are not formulated to specifically target a certain subset of DCs and/or (2) the antigen dose is not tailored to maximize the intrinsic/pre-programmed capabilities of the specific DC subset. This new and substantial departure from the status quo is expected to overcome problems that have hindered our ability to generate effective vaccines against some key pathogens.

**Graphical Abstract:**
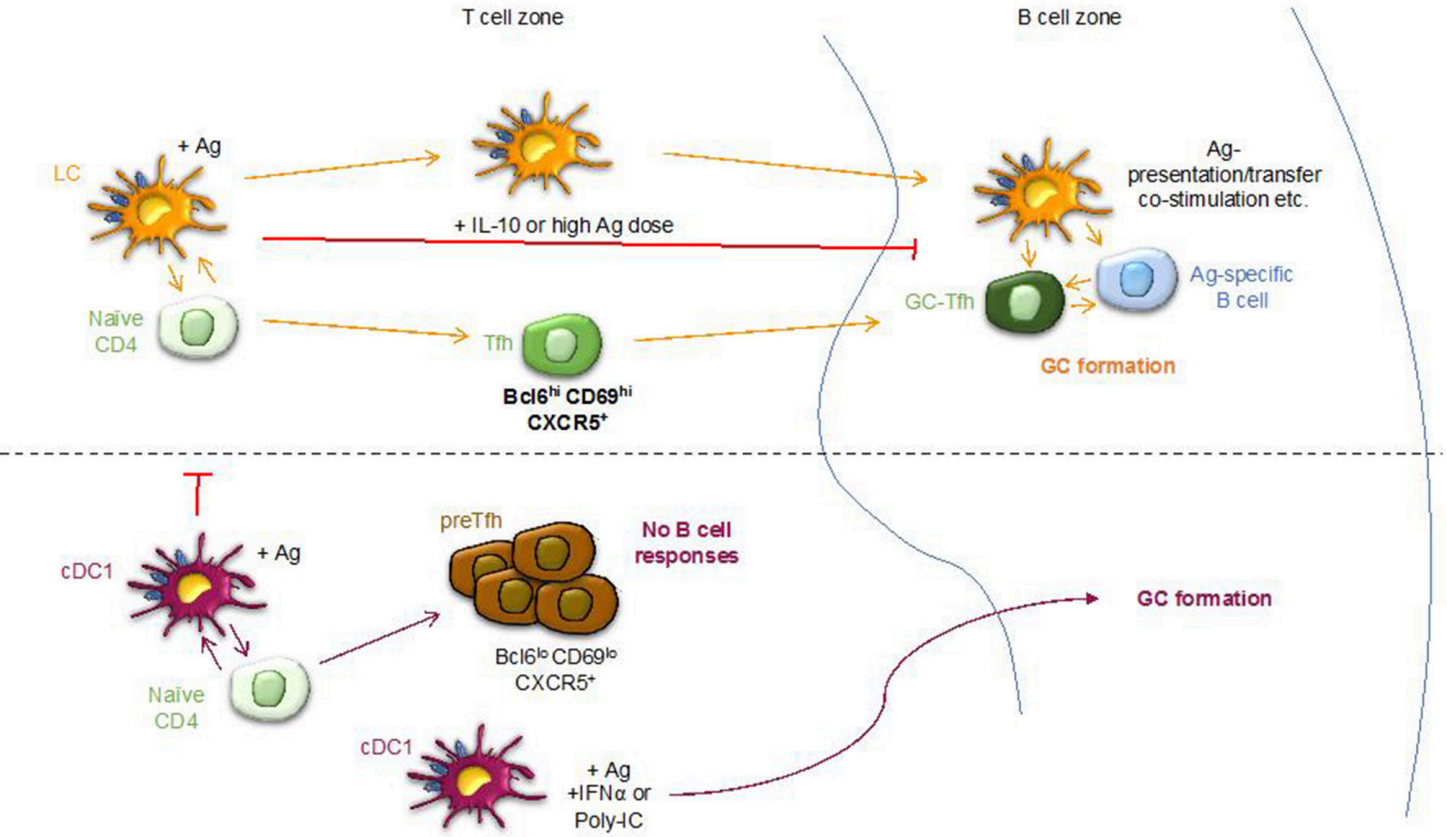
Sequential activation model. LCs and cDC1s regulate humoral immune responses by supporting the differentiation of distinct Tfh cells. LCs, unlike cDC1s support GC-dependent antibody responses in the absence of an adjuvant. LCs first induce the formation of Tfh cells and then, likely licensed by the Tfh cells, migrate to the B cell area to initiate B cell responses. The GC responses induced by LCs were inhibited by: targeted delivery of IL-10, high antigen dose and co-delivery of antigen to cDC1s. cDC1s were able to support GC-dependent humoral immune responses in inflammatory settings, in the presence of poly-IC or targeted delivery of IFNα.

## Introduction

Understanding the contribution of different immune cells to the initiation and regulation of humoral immune responses is key to the systematic development of effective vaccines. B cell responses have mostly been studied in a non-targeted fashion, using high antigen doses injected into the periphery and often combined with adjuvants (1, 2). These experimental models, unlike targeting specific antigens to certain cell types, allow extended exposure of all of the immune cells to the antigen and adjuvant and free drainage to the peripheral lymphoid organs (2). Therefore, these systems are not suitable to evaluate the contribution of a specific immune cell to humoral immune responses. The mechanism that aids the encounter of cutaneous/peripheral antigens with B cells in the lymph node is unclear (3, 4). Under steady-state conditions or during local infections, the amount of antigen entering the periphery is limited, and the local dendritic cells (DCs) might be the only cells that have access to the antigen/pathogen to initiate B cell responses.

Based on the current textbook model, the role of DCs in regulating humoral immune responses is limited to the initiation of T follicular helper cell (Tfh) development (5, 6). The possible direct effect of DCs on B cells is often disregarded or marginalized, though many studies have shown that DCs can interact and present antigen directly to B cells (7–9). For example, the transfer of *in vitro* or *in vivo* antigen-loaded DCs into naïve animals leads to generation of humoral immune responses (10). DCs, unlike B cells and macrophages, can hold on to the intact antigen in specialized mildly acidic compartments (11, 12), and through different mechanisms, can present the antigen and make it available to B cells (13). These *in vitro* interactions can lead to B cell activation and even isotype switching (14–16). DC-derived cytokines such as IL-12 and IL-6/IL-6R can promote IgM plasma cell or memory cell formation, respectively (7, 17, 18). Furthermore, through secretion of chemokines, the DCs can also coordinate the migration of B and T cells and probably help facilitate the encounter of antigen-specific T and B cells (18). Some DCs can also migrate into the B cell follicles and most likely contribute directly to regulating B cell responses (19).

Humoral immune responses can be generated through GC-dependent or -independent mechanisms. The signaling and cellular components determining which of these responses are mounted against a specific antigen are largely unknown (1, 3, 4). Tfh cells play an important role in providing help to GC-dependent B cell responses, but their possible role in GC-independent B cell responses is not fully defined (20). DCs are required for induction of Tfh cell differentiation but B cells are needed for the final maturation of the Tfh cells (5, 6, 21). Recently, the role of DC subsets in the regulation of humoral immune responses was studied using antibody vehicles to deliver antigen specifically to DCs or by using DC subset KO mice to check for the individual contribution of a specific DC subset (22, 23). The data from these models support earlier data that DCs are fundamental to fine tuning the induction and regulation of humoral immune responses. Antigen targeting to particular DC subsets leads to a diversity of B cell responses—no or weak B cell responses (23), GC-independent responses (24), and GC-dependent responses (25, 26). However, these studies did not provide mechanistic insight into how these different immune responses are generated and regulated. Furthermore, the data interpretation was also complicated by the fact that these studies targeted different DC subsets through distinct receptors, which even on the same DC type can generate diverse responses (27). To overcome this challenge and obviate complications from delivering antigen through different receptors, we used a well-established *in vivo* experimental model that allowed us to target antigen to two distinct DC subsets through the same receptor (26). Using this system, we revealed that presentation of the same antigen by LCs and cDC1s, targeted through the same receptor, led to, respectively, GC-dependent vs. null humoral immune responses. Induction of functionally distinct Tfh cells and direct effects on B cells are likely key to regulating these diverse responses. We also uncovered a negative regulatory loop by which cDC1s prevent the induction of humoral immune responses by LCs.

## Materials and Methods

### Mice

huLangerin-DTA, Batf3^−/−^, huCD40, and huLangerin-Cre-I-Aβ mice have been previously described (27–30). The transgenic mice expressing human Dectin-1/LOX-1 were generated at Taconic using the BAC clone: RP11-959D18. CD90.1 congenic TEα Rag1^−/−^ CD4 TCR transgenic mice to I-Eα_52−68_ on the C57BL/6 background were obtained from M. Jenkins (University of Minnesota). All experiments were performed with 6- to 12-week-old female and male mice. Mice were housed in microisolator cages and fed irradiated food and acidified water. The Baylor Institutional Care and Use Committee approved all mouse protocols.

### Antibodies and Reagents

Fluorochrome-conjugated antibodies to CD4 (GK1.5), CD11b (M1/70), CD11c (N418), CD25 (PC61), CD40 (3/23), CD44 (IM7), CD45.1 (A20), CD45.2 (104), CD69 (H1.2F3), CD86 (GL-1), CD103 (2E7), GL7 (GL7), CXCR5 (L138D7), PD-1 (29F.1A12), Blimp-1 (5E7), B220 (RA3-6B2), Sca-1 (D7), IgM (RMM-1), IgD (11-26c.2a), and I-A/I-E (M5/114.15.2) were purchased from BioLegend (San Diego, CA). Antibodies to CD4 (GK1.5), CD16/CD32 (2.4G2), and Live/Dead were purchased from Tonbo Biosciences (San Diego, CA). Peanut agglutinin (PNA) was purchased from GeneTex (Irvine, CA). Antibodies to CCR7 (4B12), CD90.2 (53-2.1), F4/80 (BM8), CD11b (M1/70), anti-human IgG cross-adsorbed secondary antibody, phospho-S6 (cupk43k), and Live/Dead dye were acquired from Thermo Fisher Scientific (Waltham, MA). Anti-Bcl6 (K112-91), CD90.1 (OX-7), CD38 (90), and CD138 (281-2) were purchased from BD Biosciences (Franklin lakes, NJ). Anti-S1PR1 (713412) was purchased from R&D Systems (Minneapolis, MN). Anti-langerin (929F3) was from Dendritics (Lyon, France) or purchased from BioLegend (4C7). Anti-human/anti-mouse mAb and conjugates (4C7-Eα, 4C7-doc, cohesin-IL-10, cohesin-IFNα4, anti-huCD40 and anti-huDectin-1) were generated in house, as previously described (31). All the reagents used in this study were generated using mammalian cell lines to minimize the presence of endotoxins. The average endotoxin level was 0.12 ng LPS/mg protein. Eα (I-Eα_52−68_) is a well-characterized immunodominant T cell epitope from the I-Eα MHCII molecule recognized by transgenic TEα cells in the context of I-Aβ. Poly I:C was purchased from InvivoGen (San Diego, CA).

### Flow Cytometry

Single-cell suspensions were obtained and stained, as previously described (26). All the flow cytometric plots presented in this article were pre-gated on live (using Live/Dead stain) and singlet events. Intracellular transcription factor staining was performed with the BD Bioscience Cytofix/Cytoperm kit (BD Biosciences, San Jose, CA), according to the manufacturer's instructions. Samples were analyzed on a LSRFortessa flow cytometer (BD Biosciences). Data were analyzed with FlowJo software (TreeStar; Ashland, OR).

### Endogenous B Cell Responses

Single-cell suspension of skin-draining LNs (axillary, brachial, and inguinal) from different mice were incubated *ex vivo* with AF647-conjugated huIgG4 and a cocktail of other indicated antibodies on ice for 30 min (26). After washing, the cells were analyzed on a LSRFortessa flow cytometer.

### ELISpot

The Mouse IgG/IgM Double-Color FluoroSpot kit from ImmunoSpot (Cellular Technology Limited, Cleveland, OH) was used following the recommended protocol. Briefly, plates were activated with 70% ethanol. After two washes with PBS, the plates were coated overnight at 4°C with in house generated hIgG4 antibody. Single-cell suspension of skin-draining LNs (axillary, brachial and inguinal) from different mice injected intraperitoneally with 1 μg of 4C7 were obtained 14 after immunization. Each sample was distributed in triplicate at a concentration of 1 million cells/well. The cells were then incubated at 37°C for 24 h. After washing the plates with PBS and 0.05% Tween-PBS, detection antibodies (anti-mouse IgG-FITC and IgM-Biotin) were added and incubated overnight at 4°C. The plates were then washed with 0.05% Tween-PBS before incubation with a tertiary solution (Strep-CTL-Red™ and Anti-FITC Alexa Fluor® 488) for 1 h. After the final wash, the plates were dried and sent to Cellular Technology Limited for quantification.

### Assessment of Humoral Immune Responses

Mice were injected intraperitoneally with 1 μg of 4C7 on day 0 and serum samples were obtained 14 after immunization by using BD Microtainer SST tubes (BD, Franklin Lakes, NJ) and stored at −80°C. To determine antigen-specific antibody titers, clear flat-bottom immune 96-well plates were coated with 50 μL of huIgG4 protein diluted in BupH Carbonate-Bicarbonate buffer (Thermo Fisher Scientific) at 2 μg of protein/ml and incubated overnight. After washing, plates were blocked with blocking buffer (TBS; Thermo Fisher Scientific). After blocking, the buffer was discarded and serial dilutions of serum in blocking buffer were added and incubated 2 h at 37°C. A serial dilution of a mouse anti-hIgG4 antibody (EMD Millipore) was used as a standard. After washing, plates were incubated with horseradish peroxidase (HRP)–conjugated goat anti-mouse IgG (Jackson ImmunoResearch; West Grove, PA) in blocking solution for 2 h at 37°C, washed and developed with HRP substrate (TMB Chromogen Solution: Thermo Fisher). The reaction was stopped with 1N HCl and plates were read at 450 nm using a SpectraMax Paradigm (Molecular Devices).

### Generation of Bone Marrow Chimeras

Bone marrow cells were harvested from the tibia and femur of CD45.1^+^ congenic donor WT mice as previously described (32). Recipient mice hCD40 or WT mice were irradiated with a sub-lethal dose of gamma radiation (700mGy) 6 h before the bone marrow transfer and kept on antibiotic water for 2 weeks post irradiation. 15–20 million cells were adoptively transferred into the recipient mice via the tail vein. Chimeric mice were immunized with 1 μg of anti-hCD40 8 weeks after bone marrow transfer, and the B cell responses assessed as described above. Cells from skin-draining lymph nodes were stained to determine the rate of chimerism.

### Adoptive T Cell Transfer and Analysis

T cells were adoptively transferred, as previously described (33). Briefly, six skin-draining lymph nodes (LNs), spleens, and mesenteric LNs of TEα TCR transgenic mice were disrupted through a 40 μm cell strainer, washed with sterile HBSS, and labeled with carboxyfluorescein succinimidyl ester (CFSE; Invitrogen, Carlsbad, CA), according to the manufacturer's instructions. The cells were resuspended in sterile PBS at a concentration of 1 × 10^6^ cells/ml, and 300 μl (3 × 10^5^ cells) was injected intravenously into different mouse strains. Twenty-four hours later, mice were immunized by means of intraperitoneal injection of 1 or 10 μg of 4C7-Eα, and skin-draining LNs were harvested at indicated time points.

### Histology and Cell Quantification

Skin-draining LNs were harvested at different time points after treatment/immunization and flash-frozen in OCT. Then 8 μm sections were fixed in acetone and stained with the indicated antibodies at room temperature in humidified chambers for 1 h. Then rinsed and mounted using Aqua-Poly/Mount solution and imaged immediately. The images were analyzed and the localization of the cells determined using ImageJ and Photoshop.

### qRT-PCR Analysis of Sorted DCs

LCs and cDC1s were flow sorted from naïve WT mouse LNs as previously described. mRNA was extracted from sorted DCs using a MiniPrep kit (Qiagen, Valencia, CA) and analyzed via quantitative PCR (qPCR) with TaqMan Gene Expression Assays and an ABI 7900HT (Applied Biosystems, Carlsbad, CA), as previously described (33). All Ct values were normalized to HPRT expression and are shown as 2^−Δ*Ct*^.

### Generation and *in vitro* Culture of Human LCs With Autologous T Cells

Purified cord blood CD34^+^ progenitor cells were differentiated into LCs, following a procedure adapted from Caux et al. (34). HSC were cultured in RPMI 10% FBS supplemented with SCF (25 ng/mL), TNFα (2.5 ng/mL), and GM-CSF (20 ng/mL) (Miltenyi). At day 4, a cytokine boost was added (TGFβ, 6 ng/mL; TNFα, 2.5 ng/mL; GM-CSF, 20 ng/mL). At day 8, cells were sorted on CD1a magnetic beads (Miltenyi) and further phenotyped by flow cytometry. Monocytes were isolated from PBMCs using CD14 magnetic beads (Miltenyi) and cultured in RPMI 10% FBS supplemented with GM-CSF (20 ng/mL) and IL-4 (5 ng/mL) (Miltenyi). At day 6, MoDC were collected and phenotyped by flow cytometry. CD34-LC or MoDC were then cultured with naïve CD4^+^ T cells sorted from the cord blood or PBMCs of the same donor (Miltenyi), with a LC:T ratio of 1:4. Four days later, the CD4^+^ T population was phenotyped for Tfh markers by flow cytometry.

### Human Antibodies and Reagents

The purity of *in vitro* differentiated LCs was determined by flow cytometry (LSRII Flow Cytometer; BD Biosciences) using CD45-APC-H7 (2D1, BD Pharmingen), HLA-DR-AF700 (G46-6, BDPharmingen), CD1a-PerCP-Cy5.5 (HI149, BioLegend), CD11c-PCF594 (B-ly6, BD Horizon), CD1c-PE-Cy7 (L161, BioLegend), CD207-PE (10E2, BioLegend). MoDC differentiation was determined by flow cytometry using HLA-DR-AF700 (G46-6), CD11c-PCF594 (B-ly6), CD14-V450 (MφP9, BD Horizon), DC-SIGN (CD209)-FITC (DCN46, BD Pharmingen) antibodies. Differentiation of naïve CD4^+^ T cells into Tfh was determined by flow cytometry using CD3-BV605 (SK7, BD Horizon), CD4-PCF-594 (RPA-T4, BD Horizon); CD45RO-BV650 (UCHL1, BD Horizon), CXCR5 (CD185)-BB515 (RF8B2, BD Horizon), PD1 (CD279)-BV421 (EH12.1, BD Horizon), Bcl-6-PE (K112-91; BD Pharmingen), ICOS (CD278)-PE-Cy7 (ISA-3, eBiosciences). Cell viability was determined using Live-dead Aqua or Near-IR (Thermo Fisher Scientific). Intracellular staining of T cells was performed using FoxP3/Transcription factor staining buffer set (eBioscience).

### NHP Studies

Adult male cynomolgus macaques (*Macaca fascicularis*) imported from Mauritius and weighing 4–8 kg were housed in Commissariat à l'Energie Atomique facilities (accreditation B 92-032-02) and handled (investigator accreditation RLG, B 92-073; FM, C 92-241) in accordance with European guidelines for NHP care (EU Directive N 2010/63/EU). Before the start of the study, the animals were tested and found to be seronegative for several pathogens (SIV, simian T lymphotropic virus, filovirus, hepatitis B virus, herpes B, and measles). Animals were sedated with ketamine chlorhydrate (10–20 mg/kg; Rhone-Mérieux, Lyon, France) during handling. Animals were euthanized by sedation with ketamine, followed by i.v. injection of a lethal dose of sodium pentobarbital. The regional animal care and use committee (Comité Régional d'Ethique Ile de France Sud, reference 12-013) and the Baylor Research Institute Animal Care and Use Committee (IACUC reference A10-015) reviewed and approved this study.

### Immunizations

Groups of three to six cynomolgus macaques underwent inoculation at weeks 0, 6, and 15, with 1 mL (10 i.d. injections of 100 μl) indicated vaccine preparation. Each preparation contained 62.5 μg HIV Gag p24 protein, corresponding to 250 μg total protein when associated with Abs in fusion proteins. Sera were collected from vaccinated animals for the titration of Gag-specific Abs with the Gag-specific IgG Ab ELISA, as described (35).

### Statistical Analysis

Differences between 2 data sets were analyzed by using the 2-tailed Student *t*-test and ANOVA with GraphPad Prism software (GraphPad Software, La Jolla, CA).

## Results

### Steady State LCs, but not cDC1s can Support the Formation of GC-Dependent Antibody Responses; cDC1s Prevent LCs From Inducing Humoral Immune Responses

**Figure 1:**
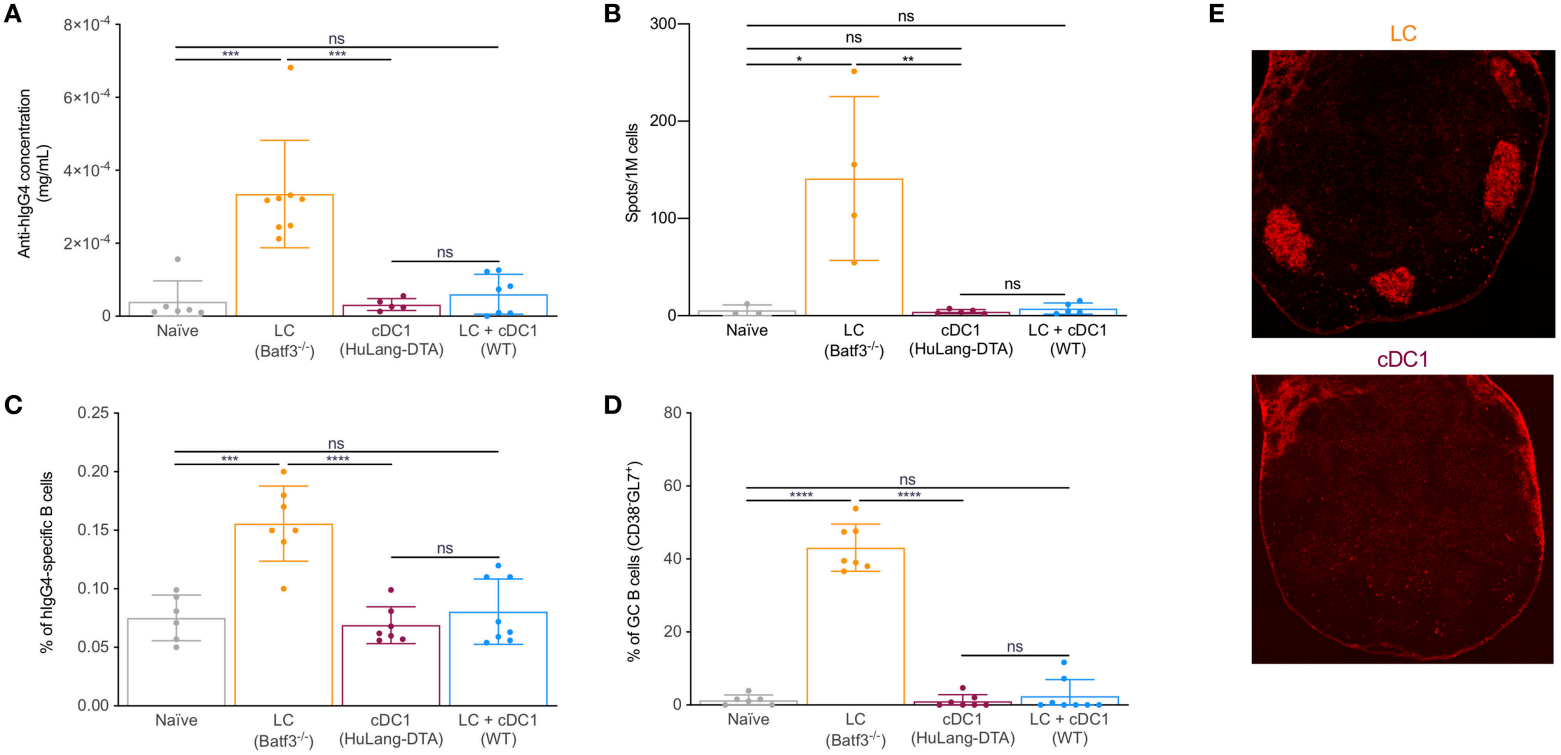
Steady state LCs, but not cDC1s can support the formation of GC-dependent antibody responses; cDC1s prevent LCs from inducing humoral immune responses. Mice were immunized with 1 μg of 4C7 (hIgG4 core) through the indicated DC subsets and the anti-hIgG4 responses induced were assessed 14 days later by: **(A)** ELISA (serum), **(B)** ELISpot (LNs), **(C)** Flow cytometry (LNs, % of hIgG4-specific B cells), **(D)** Flow cytometry % GC B cells (CD38^+^GL7^−^), and (**E)** histology. Skin draining LNs harvested from mice immunized through LCs or cDC1s were harvested at day 14 and stained with PNA to highlight GCs. Each dot represents a separate mouse. Data from at least 3 different experiments were pooled. ***p < 0.001, ****p < 0.0001, ns = not significant.

We previously found that LCs, but not cDC1s, were efficient drivers of humoral immune responses against foreign antigen in the absence of exogenous adjuvants (26). Here, we sought to further characterize the regulation of humoral immune responses induced by these DC subsets. For this purpose, we used a well-characterized antibody-targeting system that employs anti-Langerin antibody to specifically deliver antigens to Langerin-expressing LCs and cDC1s localized to skin-draining LNs and skin (26, 33). Langerin in mouse skin and LNs is expressed at high levels by epidermal LCs and dermal cDC1s, also called XCR-1 DCs or CD103^+^ DCs (36). To limit antigen delivery to LCs or cDC1s, we used Batf3^−/−^ mice (specifically lacking cDC1s) (29) or huLangerin-DTA mice (specifically lacking LCs) (28), respectively (26). In WT mice, the anti-Langerin antibody targets both LCs and cDC1s (26). On figures, to clarify the study designs we display the DC subset (s) driving the response and in parenthesis the mouse strains used. The DC subset knock-out mice along with WT littermate controls, were immunized intraperitoneally with 1 μg of human chimeric anti-Langerin (4C7, hIgG4) antibody, and the primary humoral immune responses mounted against the human portion of 4C7 were assessed by ELISA, ELISpot, flow cytometry, and histology 14 days later. ELISA showed robust induction of mouse IgGs against the targeting construct by LCs, but not when cDC1s were targeted (Figure 1A). More importantly, in WT mice where both LCs and cDC1s are targeted the mice again failed to generate significant amounts of antibodies (Figure 1A). ELISpot assays on LN cells revealed the presence of hIgG4-specific IgG secreting cells in mice immunized through LCs, but not through cDC1s or when both DCs were targeted (Figure 1B). Fluorochrome-labeled hIgG4 allows tracking of the antigen-specific B cell responses [(26) and Supplementary Figure 1A]. Flow cytometry analysis of antigen-specific B cells from skin-draining LNs showed increased numbers of hIgG4-specific B cells (Figure 1C) concurrent with robust germinal center (GC) (Figure 1D) induction by LCs, but not by cDC1s. No GC-induction was observed when the antigen was delivered to both LCs and cDC1s (Figure 1D). PNA-histology confirmed the presence of GCs when LCs were targeted and a complete absence when antigen was delivered to cDC1s alone (Figure 1E) or in combination with LCs (data not shown). Thus, LCs, unlike cDC1s, promote GC-dependent humoral immune responses in the absence of any adjuvants and the delivery of antigen to both LCs and cDC1s is detrimental to the LC-induced antibody responses.

To test whether the induction of GC-dependent humoral immune responses by LCs is limited to Langerin targeting, we generated a new mouse model. We irradiated human CD40 (hCD40) mice (27) and reconstituted them with congenically marked CD45.1^+^ WT bone marrow cells. Since LCs are radiation resistant, the only cells that will express hCD40 after radiation and reconstitution are the LCs (Supplementary Figure 1B). Eight weeks after reconstitution, the mice were injected with 1 μg of human chimeric anti-hCD40 [this mAb does not cross-react with mouse CD40; (37)]. Fourteen days after immunization, we found that targeting antigen to LCs through CD40 also led to induction of GC-dependent humoral immune responses (Supplementary Figure 1B). Anti-hCD40 injection control chimeras did not lead to GC responses (Supplementary Figure 1B). Thus, LC-induced GC responses in steady state are not limited to Langerin targeting.

In order to determine whether the inability of cDC1s to drive humoral immune responses is limited to Langerin targeting or not, we took advantage of a newly generated human Dectin-1 BAC transgenic mouse model. These transgenic mice were generated at Taconic by using a BAC containing the human gene coding for Dectin-1 and LOX-1. We found that the expression of human Dectin-1 in these mice was limited to cDC1s (Supplementary Figure 1C). Targeting the cDC1s with human chimeric anti-human Dectin-1 antibody [this mAb does not cross-react with mouse Dectin-1; (38)] led to no GC responses (Supplementary Figure 1C). Thus, these data suggest that steady state cDC1s, independently of the receptor targeted do not support the formation of antibody responses.

### LCs can Migrate to the B Cell Zone and Activate B Cells

**Figure 2:**
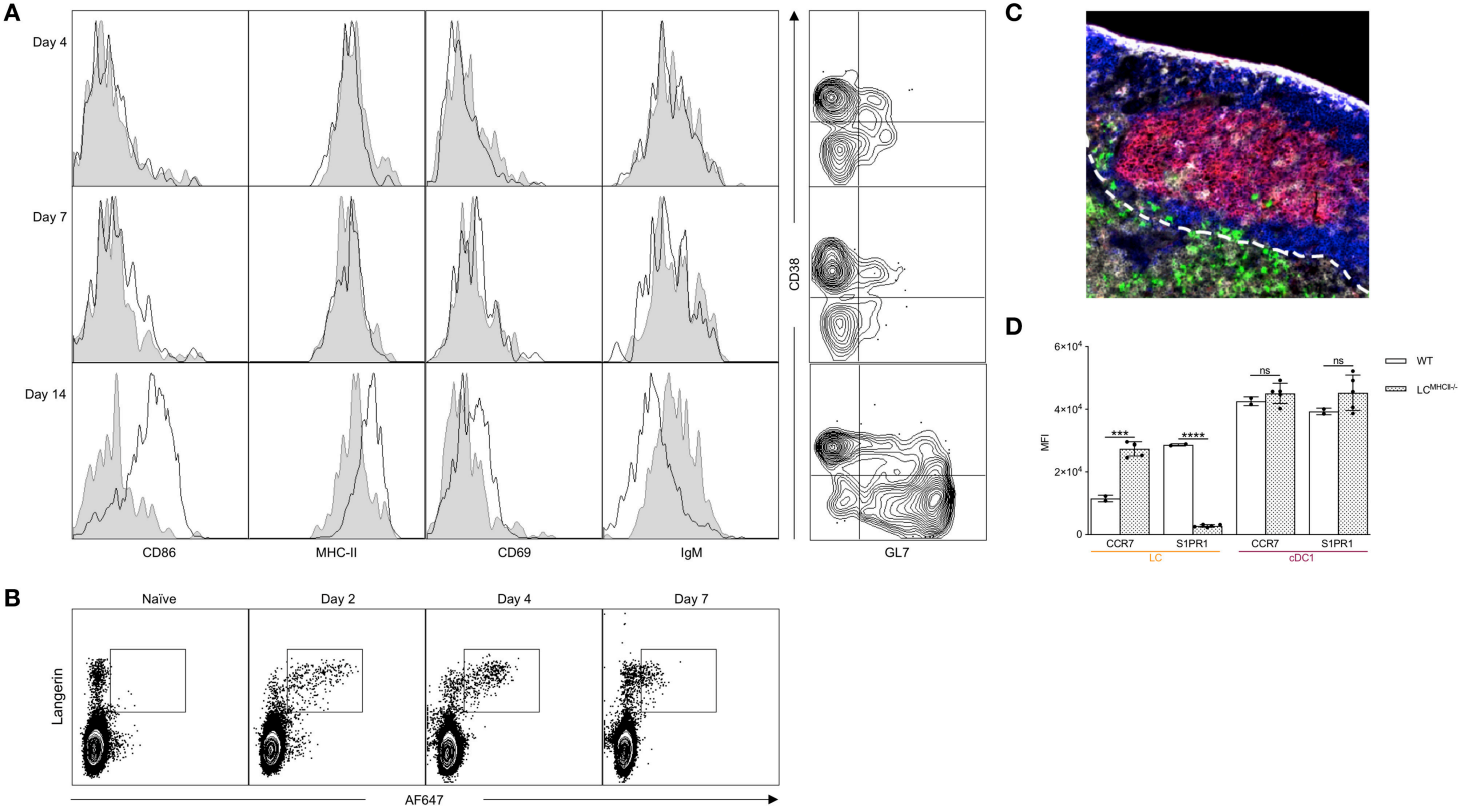
LCs can migrate to the B cell zone and activate B cells. **(A)** Left: Mice were immunized through LCs and the hIgG4-specific B cell phenotype determined by flow cytometry at the indicated timepoints. Black lines indicate immunized mice, while gray shaded histograms are from naïve mice. Right: The phenotype of the hIgG4-specific B cells displayed using CD38 and GL7 markers. One representative experiment out of three is shown. **(B)** WT mice were injected with 10 μg of AF647-labeled 4C7 antibody. At the indicated timepoints, the AF647 signal in LN Langerin^+^ cells (LC and cDC1) was determined by flow cytometry. One representative experiment out of three is shown. **(C)** Localization of LCs 14 days after immunization in a Batf3^−/−^ mouse (red: PNA, blue: B220, green: Langerin, and gray: CD4). One representative experiment out of three is shown. **(D)** CCR7 and S1PR1 expression in LCs and cDC1s in WT mice and in mice in which LCs lack MHC-II. Each dot represents an individual mouse. One representative experiment out two is shown. ***p < 0.001, ****p < 0.0001, ns = not significant.

We previously showed that isotype injected mice, similarly to WT mice injected with anti-huLangerin, did not generate humoral immune responses against the targeting construct (26). These data suggest that direct acquisition of antigen by the antigen-specific B cells is not sufficient to initiate humoral immune responses in our targeting model. Thus, we hypothesized that in order for the B cells to have access to the antigen and get activated, the LCs assume an active role. The B cells will therefore present the antigen to pre-Tfh/Tfh cells in order to receive the necessary help for isotype switching, memory- and plasma cell formation. Thus, next, we sought to determine the kinetics of the B cell responses induced by LCs. We targeted LCs with 1 μg of 4C7, and the mice were euthanized at different timepoints and the antigen-specific B cell responses characterized by flow cytometry. We found that the antigen-specific B cells showed no activation in the first 7 days post immunization (Figure 2A). By the end of the second week post immunization the antigen-specific B cells upregulated MHC-II, CD69, CD86 (Figure 2A) and downregulated IgM and formed B cells with GC phenotype (Figure 2A). Concomitant with this, we found that LCs were able to retain detectable amounts of intact antigen for at least seven days (Figure 2B); and some LCs could be found in the B cell follicles including GCs by day 14 (Figure 2C). Interestingly, LCs showed upregulation of CCR7 and complete loss of S1PR1 in the absence of cognate interaction with CD4^+^ T cells (Figure 2D), and were unable to drive humoral immune responses (26). These data, in the light of our previous observations that the Tfh cell responses initiated by LCs peak at day 4 (transgenic) or 7 (endogenous) (26), suggest the existence of a sequential activation model for the development of humoral immune responses. We speculate that LCs first initiate the development of Tfh cells and when the T cell help is present, the T cell-licensed LCs deliver the intact antigen to the B cells.

### IL-10 and High Antigen Dose Limit LCs' Ability to Induce GC-Dependent Humoral Immune Responses

**Figure 3:**
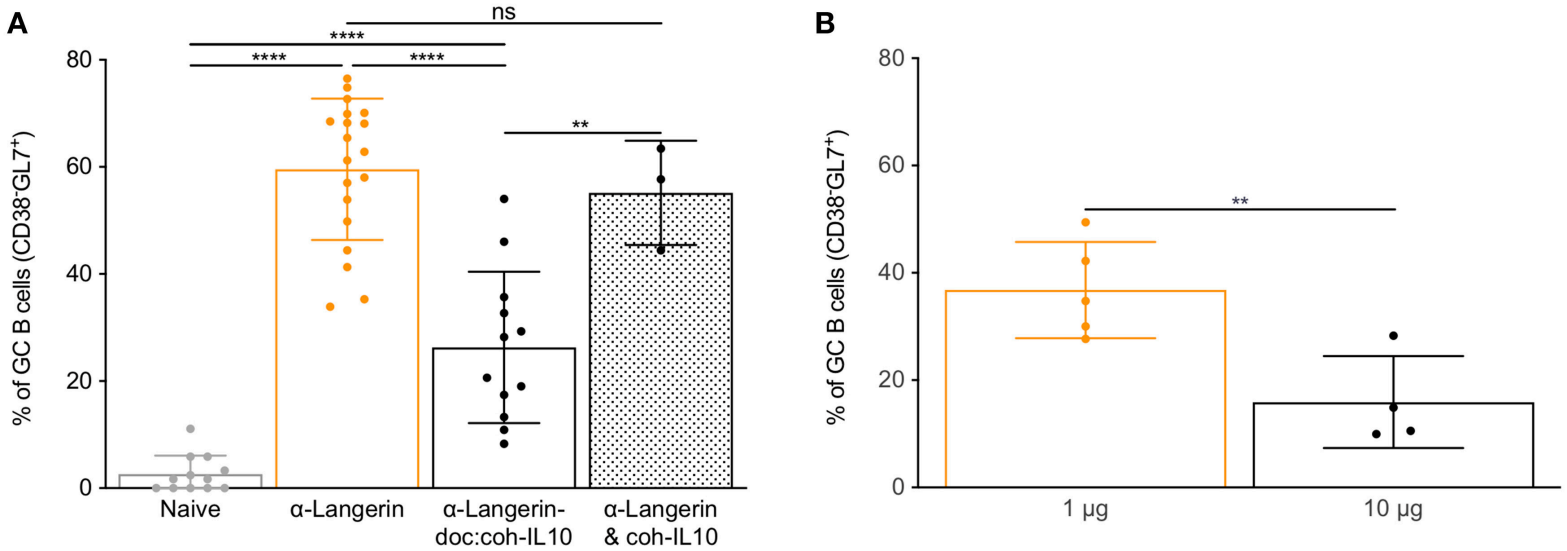
IL-10 and high antigen dose limit LC's ability to induce GC responses. **(A)** LCs were targeted with α-langerin antibody in the absence or presence of IL-10. The IL-10 was either directly linked to the antibody (doc:coh-IL-10) or just mixed with the antibody (& coh-IL-10). Fourteen days later the GC responses were determined by flow cytometer. Data from multiple experiments were pooled. Each dot represents a separate mouse. **p < 0.01, ****p < 0.0001, ns, not significant. **(B)** LCs were targeted with either 1 or 10 μg of antibodies. Fourteen days later the GC responses characterized using flow cytometry. Data from one representative experiment out of two is shown. Each dot represents a separate mouse. **p < 0.01.

IL-10 is a pleiotropic cytokine with high clinical potential that plays an important role in regulating both T and B cell responses (39). Selective IL-10 signaling blockade in DCs leads to enhanced humoral immune responses (40). To define the effects of IL-10 on LC-induced humoral immune responses, we generated new targeting constructs. For the generation of these constructs, we used a previously described technology that relies on the high-affinity interactions between dockerin (doc) and cohesin (coh) (31). Dockerin was fused to the heavy chain of the 4C7 antibody, and the N-terminus of IL-10 was attached to the C-terminus of cohesin. We targeted LCs with 1 μg of 4C7, 4C7-doc:coh-IL-10 (non-covalent complex), or 4C7 plus free coh-IL-10 (control, non-complex). Fourteen days later, we found that IL-10 delivery by 4C7 targeting led to significant decrease of GC responses (Figure 3A). IL-10 did not inhibit the binding of 4C7 to Langerin (Supplementary Figure 2A). Free coh-IL-10 co-injected with 4C7 (without doc) did not have a significant effect on humoral immune responses (Figure 3A). IL-10 delivery to LCs, however, did not lead to significant decrease of total serum antibody levels (Supplementary Figures 2B,C). Thus, LC-induced GC responses can be modulated by IL-10 signaling.

Humoral immune responses show antigen dose dependency (41–43). To test the effect of antigen dose on LC-induced B cell responses, we targeted LCs with low (1 μg) or high (10 μg) doses of 4C7. We found that LCs loaded with the high antigen dose were less efficient at driving humoral immune responses (Figure 3B and Supplementary Figure 2D). Thus, high antigen dose negatively affects humoral immune responses induced by LCs, probably by limiting the formation of GC Tfh cells (26).

### LCs, Unlike cDC1s, Promote Tfh Cells With GC Phenotype

**Figure 4:**
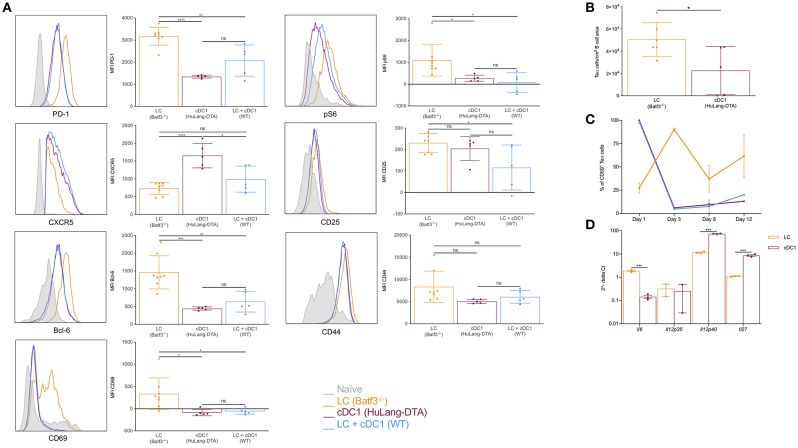
LCs, unlike cDC1s, promote Tfh cells with GC phenotype; cDC1s might outcompete the LCs on T cell activation levels. **(A)** Mice were transferred with transgenic TEα cells and immunized through the indicated DC subsets with 1 μg of 4C7-Eα. The phenotype of the TEα cells was assessed by flow cytometry 4 days later, at the peak of the response. Representative flow histograms followed with compiled MFI data from multiple mice. Data from two experiments are shown. Each dot represents a separate mouse. **(B)** As in **(A)**, but LNs were harvested and the number of TEα cells in the B cell area determined using multicolor histology and ImageJ. Data from one representative experiment out of two is shown. Each dot represents a separate mouse. **(C)** Similar to **(A)**, but the LNs were harvested at the indicated timepoints and the activation status of the TEα cells determined based on CD69 expression using flow cytometry. Data from one representative experiment out of two is shown. *N* = 3. (**D)** Steady state LCs and cDC1s were flow sorted from LNs and their cytokine profile determined by qRT-PCR. Expression levels were normalized to HPRT. Each dot represents a separate mouse. *p < 0.05, **p < 0.01, ***p < 0.001, ns = not significant.

DCs regulate humoral immune responses by initiating the development of Tfh cells. Previously we showed that LCs and cDC1s promoted PD-1^+^CXCR5^+^ Tfh cells upon Langerin targeting (26). Since LCs, unlike cDC1s, were able to support GC-dependent humoral immune responses, we hypothesized that LCs will support the development of Tfh cells with a GC-Tfh phenotype. To further characterize the CD4^+^ T cell responses induced by these two DCs, we adoptively transferred CFSE-labeled transgenic TEα cells into mice lacking LCs, cDC1s or WT littermate control mice. We immunized the mice with 1 μg of 4C7-Eα and analyzed the expression of common Tfh/GC-Tfh cell (PD-1, CXCR5 and Bcl-6) and activation markers (CD25, CD44, CD69, and pS6) by the TEα cells at day 4 (peak of the response) (26) using flow cytometry. In concordance with previously published data (26), we found that cDC1s induced a more robust expansion of the antigen-specific CD4^+^ T cells, than the LCs (Supplementary Figures 3A,B). Both DC subsets were able to induce PD-1/CXCR5 double positive cells (Supplementary Figure 3A). In addition, we show that targeting both LCs and cDC1s lead to comparable responses to those from targeting cDC1s alone (Supplementary Figures 3A,B). We further found that TEα cells educated by LCs showed higher levels of CD69, pS6, PD-1, and Bcl-6 compared to cDC1s or the combination of LCs and cDC1s, but expressed lower levels of CXCR5 (Figure 4A). Histology also revealed that the TEα cells activated by LCs were more efficient in migrating into the B cell area than the ones induced by the cDC1s (Figure 4B). Thus, these data support the notion that LCs and cDC1s promote Tfh cells with distinct phenotypes.

Antigen presentation by cDC1s alone or in combination with LCs led to similar T cell phenotypes (Figure 4A). These data prompted us to hypothesize that cDC1s might have superior T cell activation capacity and outcompete LCs. To determine whether LCs and cDC1s differ in T cell activation capacity we adoptively transferred CFSE-labeled transgenic TEα cells into mice lacking LCs, cDC1s or WT littermate control mice and immunized them with 1 μg of 4C7-Eα. At different time points the LNs were harvested and the activation of TEα cells were monitored by CD69 expression. We found that cDC1s activated T cells faster than LCs. As early as day 1 after immunization close to 100% of the TEα cells upregulated CD69 upon interaction with cDC1s, and they showed significant cell proliferation by day 3 with a concomitant drop in CD69 levels (Figure 4C). The T cell activation by LCs presented a significantly different kinetic. It took 3 days for the LCs to activate majority of the TEα cells (Figure 4C) and the T cell expansion was still minimal compared to the one induced by cDC1s (Supplementary Figures 3A,B). When both DC presented antigen, the T cell responses followed similar kinetics to one observed when cDC1s were the sole presenters (Figure 4C). Thus, faster T cell activation capacity of cDC1s might confer them with a competitive advantage over LCs and could constitute a potential mechanism by which cDC1s prevent LCs from inducing antibody responses.

In mice, IL-6 is a pro-Tfh cytokine, IL-12 inhibits Tfh formation, and IL-27 can sustain Tfh proliferation (44, 45). To test whether LCs and cDC1s can make Tfh cell-promoting/suppressing cytokines, we sorted these DCs subsets from steady state skin-draining LNs and performed qRT-PCR for *Il6, Il12p35, Il12p40*, and *Il27*. We found that LCs contained significantly higher levels of IL-6 mRNA, but they were less efficient at synthesizing IL-12 and IL-27 mRNAs than cDC1s (Figure 4D). The expression of these cytokines are controlled by the Bcl-6/Blimp-1 transcription factor duo (45). Interestingly, we found that Blimp-1 expression, which promotes IL-12 production, was significantly higher in cDC1s (Supplementary Figure 3C), while LCs expressed relatively higher levels of Bcl-6 (Supplementary Figure 3C), which supports IL-6 expression. Thus, we speculate that differential cytokine expression regulated by transcription factors might contribute to the distinct ability of LCs in induction of GC Tfh cells.

### Inflammatory Signal Enables cDC1s to Drive GC-Dependent Humoral Immune Responses

**Figure 5:**
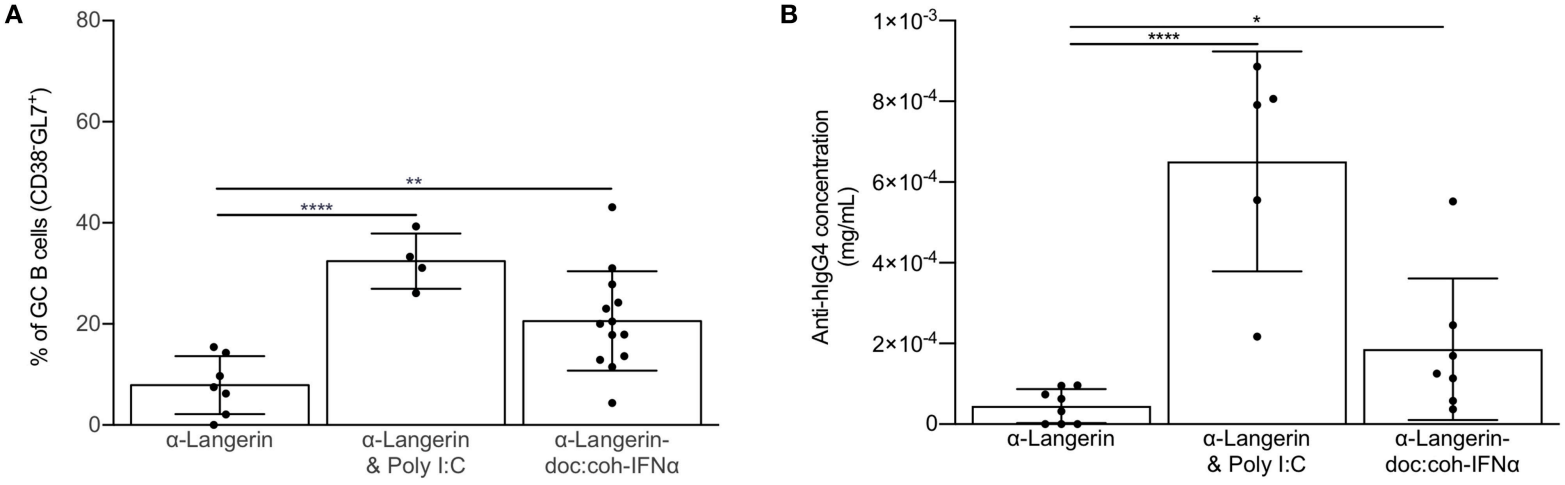
Inflammatory signal enables cDC1s to drive GC-dependent humoral immune responses. **(A)** cDC1s were targeted in the absence or presence of the indicated adjuvant or inflammatory cytokine and the percentage of hIgG4-specific germinal center B cells assessed by flow cytometry 14 days after immunization. **(B)** As in **(A)** except the anti-hIgG4 responses were assessed in the serum using ELISA. Data from multiple experiments pooled. Each dot represents a separate mouse. *p < 0.05, **p < 0.01, ****p < 0.0001.

In most cases induction of humoral immune responses requires the use of adjuvants (3). Thus, we next assessed whether co-delivery of antigen with commonly used adjuvant, poly I:C, can potentiate humoral immune responses through cDC1s. We immunized mice lacking LCs or WT littermate control mice with 4C7 or 4C7 mixed with 100 μg of poly I:C. We found that poly I:C enabled cDC1s to induce GC responses (Figure 5). Poly I:C's main mechanism of action is through induction of IFNα, which plays an important role in regulating DC biology (46). Thus, we tested whether IFNα delivered directly linked to the antigen could enable cDC1s to drive GC-dependent humoral immune responses. To test this, we immunized mice lacking LCs or WT littermate control mice with 4C7 or 4C7-doc:coh-IFNα. Fourteen days later we indeed found that delivery of IFNα enabled cDC1s to drive GC responses both in presence or absence of LCs (Figure 5). The induction of GC responses by cDC1s was IFNα dose-dependent (data not shown). Thus, these data suggest that cDC1s can drive GC-dependent humoral immune responses in an inflammatory setting.

### Human LCs Promote the Formation of Tfh Cells

**Figure 6:**
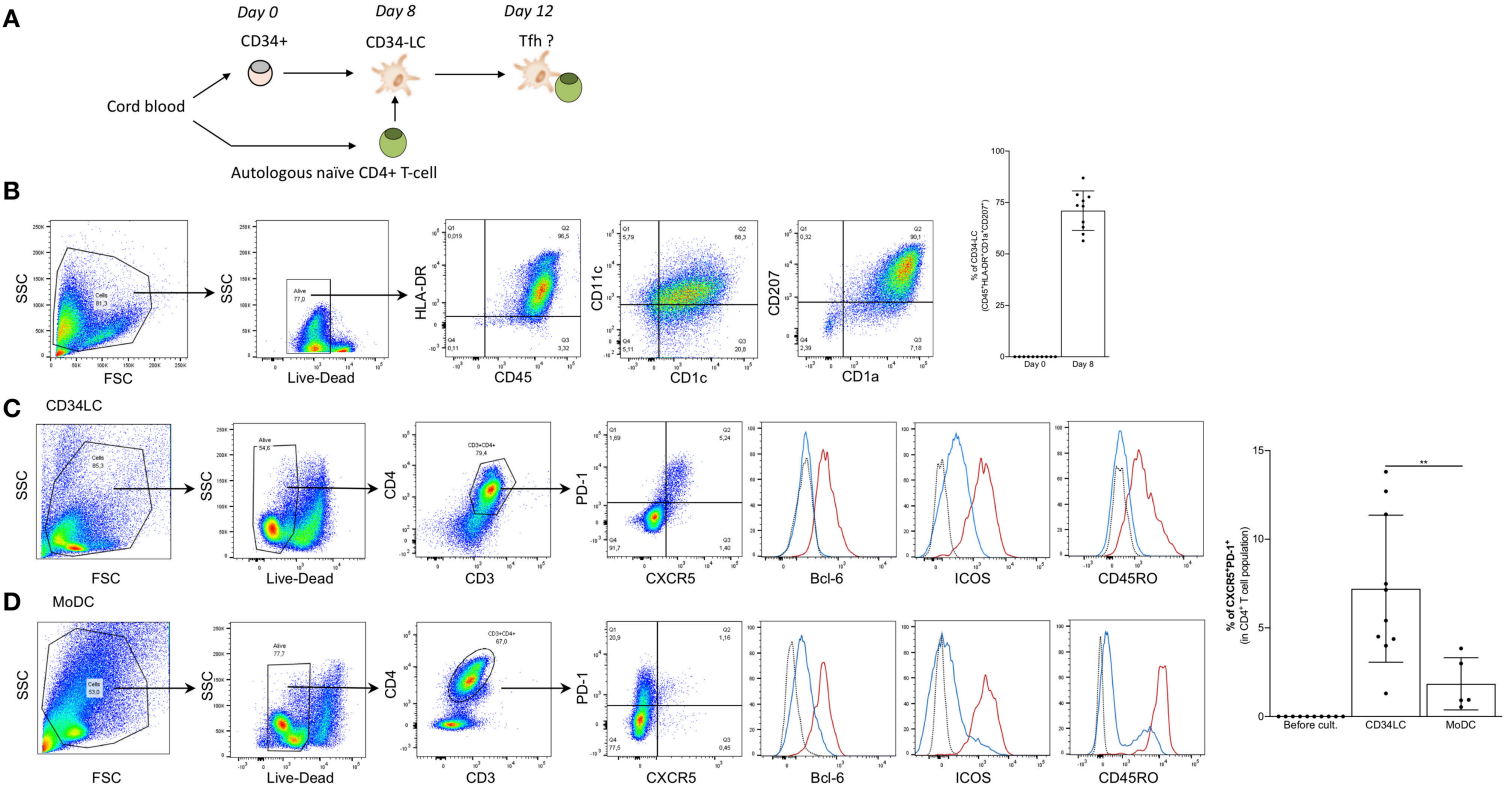
Human LCs promote the formation of Tfh cells. **(A)** Schematics of the procedure. **(B)** Phenotype of CD34-LCs at day 8. Human LCs differentiated from CD34^+^ HSCs were sorted for CD1a expression and checked for Langerin (CD207) and other DC phenotypical markers. Right: Percentages of CD1a^+^ CD207^+^ double positive LCs for 10 donors are shown. **(C)** Autologous naïve CD4^+^ T cell were added to CD34-LCs at day 8. Four days later, the CD4^+^ T cells were analyzed for the indicated markers. The red line indicates CXCR5^+^PD-1^+^, blue line CXCR5^−^PD-1^−^ cells and the dotted line represents isotype control. **(D)** Same as in **(C)** but MoDCs were used instead of CD34-LCs. Percentages of CXCR5^+^ PD-1^+^ CD4^+^ T-cells induced by CD34-LCs and moDCs are indicated for 10 and 5 donors, respectively. **p < 0.005.

The role of human LCs in the induction of Tregs in steady state and Th17 upon *Candida* infection has been documented (47). Furthermore, Penel-Sotirakis et al. reported that freshly emigrated LCs in an allogeneic setting were able to support the formation of IL-21 producing CD4^+^ T cells (48). Since IL-21 is often associated with Tfh cells (21) it is likely that the reported IL-21 producing cells were Tfh-like cells. However, to further characterize the capacity of human LCs to drive Tfh cell responses, and to test whether they are capable of inducing Tfh cells in an autologous system we developed an *in vitro* model. For this purpose, LCs were differentiated from cord blood-derived CD34^+^ HSCs and co-cultured with autologous naïve CD4^+^ T cells (Figure 6A). *In vitro* CD34-derived LCs (CD34-LC) exhibit characteristic markers of LCs from human skin (CD45^+^ HLA-DR^+^ CD11c^+^, CD1c^+^, CD207^+^, and high expression of CD1a) (Figure 6B). Of note, *in vitro* LCs remained immature (CD83, CD86, HLA-I, and -II low). We observed that LCs could induce the differentiation of naïve CD4 T cells into GC Tfh-like cells that were characterized by expression of PD-1, CXCR5, Bcl-6, ICOS, and the CD45RO memory phenotype (7.2 ± 4% of total CD3^+^ CD4^+^ T cells) (Figure 6C). In the same culture conditions, the monocyte-derived DCs did not promote significant Tfh cell differentiation (Figure 6D). Thus, these data suggest that human LCs can support Tfh cell differentiation.

### Non-Human Primate (NHP) LCs Targeted With Langerin Promote Protective Antibody Responses, but Fail to Induce CD8^+^ T Cell Responses

**Figure 7:**
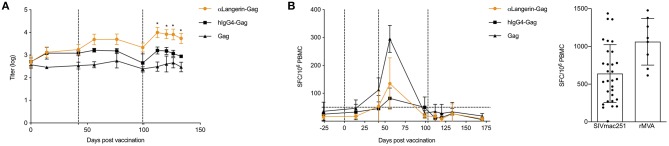
Non-human primate (NHP) LCs targeted through langerin promote antibody responses, but fail to induce CD8^+^ T cell responses. **(A)** Animals were bled at the indicated time points and the serum anti-Gag levels determined by ELISA. Vertical dotted lines indicate the time of immunizations. **(B)** Gag-specific IFNγ ELISpot results on PBMCs harvested at the indicated time points are shown. Vertical dotted lines indicate the time of immunizations. Horizontal dot line indicates background level. On the right, positive controls for the ELISpot assay. *p < 0.05.

A pioneering *in vitro* study of human LCs suggested that they were involved in triggering the CTL response rather than the humoral response (49). However, LCs may not be in the same state *in vivo* and may be influenced differently by the microenvironment. Our previous findings showed that anti-Langerin targeting of HIV-1 gag to LCs in NHPs led to antibody responses (35). To test whether anti-Langerin targeting can also induce CD8^+^ T cell responses, we repeated our previous experiments and measured both humoral immune responses and CD8^+^ T cell responses. Three i.d. injections of the anti–Langerin-gag vaccine (250 μg each) at weeks 0, 6, and 15 were sufficient to induce a significant anti-gag antibody response in NHPs (Figure 7A). Remarkably, the induction of this response did not require the use of an adjuvant, similar to what we observed in our mouse data. By contrast, animals injected with HIV-1 gag protein alone or with the IgG4-gag isotype control displayed significantly weaker responses. Therefore, antigen targeting to skin LCs *in vivo* significantly improves their ability to prime antigen-specific responses in a relevant model for human vaccine testing. The intermediate antibody response induced by the IgG4-gag could be attributed to a slight binding of gag to APCs (Figure 7A) or to possible slower clearance due to its IgG character. Interestingly, we were not able to detect any significant CD8^+^ T cell responses using ELISpot and ICS (Figure 7B). Thus, these data are in concordance with *in vivo* mouse data. They suggest that steady state LCs are potent inducers of humoral immune responses but fail to efficiently cross-present antigens to CD8^+^ T cells.

## Discussion

Herein, we provide experimental evidence that steady state LCs and cDC1s promote, respectively, GC-dependent versus null humoral immune responses. Some of the LCs could migrate into the B cell area including B cell follicles and GCs. The GC-dependent humoral immune responses induced by LCs were not limited to Langerin targeting, and could be suppressed by IL-10, high antigen dose and by cDC1s. The cDC1s were more efficient at T cell activation than LCs, which might be one of the mechanisms by which cDC1s control the humoral immune responses induced by LCs. LC-induced Tfh cells were CD69^+^, Bcl-6^high^ (GC Tfh) that efficiently migrated to the B cells area, whereas, the ones induced by cDC1s or the combination of LCs and cDC1s were CD69^−^ Bcl-6^low^ (pre-Tfh) and localized mainly to the T cell zone. We also found that poly I:C adjuvant and IFNα delivery to cDC1s enabled them to drive GC-dependent humoral immune responses. Furthermore, CD34^+^ HSC-derived human LCs, but not monocyte-derived DCs, promoted GC Tfh cell formation in an *in vitro* autologous system. Finally, in agreement with *in vivo* mouse studies (33, 50, 51), we showed in NHP studies that targeting LCs in the absence of an adjuvant leads to antibody responses but no CD8^+^ T cell responses.

The exact mechanism by which LCs and cDC1s induce Tfh cells with different phenotypes remains to be determined. Both cell types were targeted through the same receptor to minimize caveats associated with signaling pathways and intracellular antigen processing and routing. Antigen dose, precursor numbers and TCR affinity are known to affect Th differentiation (52–55). However, in our case, the number of transgenic TEα cells is controlled, and the cells carry the same TCRs. In the LNs the ratio of LCs and cDC1s in our mouse colony is ~ 1:1, and the two subsets of DCs acquire similar amounts of antigens (Supplemental Figure 4). Thus, in this case the dwelling time of the CD4^+^ T cells on the surface of DCs regulated by synapse strength might become more relevant in determining Th differentiation (53, 56). We discovered that cDC1s intrinsically are more potent CD4^+^ T cell activators than LCs, which could have contributed to the differences seen in Th phenotype and differentiation. The cDC1s also express higher levels of LFA-1 (CD11a; Supplemental Figure 5) than LCs, which could lead to stronger synapse formation between T cells and DCs and thus extended and stronger TCR stimulation (57). Interestingly, LCs loaded with higher doses of antigen induced Tfh cells that had similar phenotypes to the ones induced by cDC1s (26), highlighting also the importance of peptide/MHC load on DCs in determining Th phenotype. These two subsets of DCs also showed distinct cytokine profiles that could affect Th polarization, and their role in this remains to be explored. While LCs express high levels of IL-6 transcripts, cDC1s contain significantly more IL-12 transcript than LCs. IL-6 is a key polarizing cytokine for mouse Tfh/GC-Tfh cells (45). IL-12 plays an important role in the development of human Tfh cells (58), but it is considered an inhibitory cytokine for mouse Tfh cells (45). The distinct cytokine profile of LCs and cDC1s might be due to the differential expression of Bcl-6 and Blimp-1 transcription factors. Blimp-1, which is highly expressed in cDC1s, can directly suppress the production of IL-6 and INFα by these cells (59). Interestingly, we found that delivering IFNα specifically to cDC1s, a cytokine known to increase IL-6 production and inhibit IL-12 in DCs (46), enabled the cDC1s to promote GC-dependent humoral immune responses. These data also suggest that inflammatory conditions that release IFNα (such as viral infections) or other adjuvants can enable DC subsets, that are otherwise incapable to drive GC-dependent humoral immune responses. IL-10 signaling blockade in DCs leads to enhanced IL-6 production and higher Tfh cell numbers (40). Our finding that delivery of IL-10 to LCs can inhibit GC responses is in accordance with this and could further support the role of IL-6 in the generation of GC-supporting Tfh cells. However, further studies are needed to dissect the role of DC-derived IL-6 in regulating humoral immune responses. These data also suggest that targeted delivery of cytokines to DCs can be used to modulate immune responses, which harbor important therapeutic potentials. Targeting inhibitory cytokines to DCs could be used to promote tolerance in organ transplantation, autoimmune diseases such as lupus and psoriasis and type 1 diabetes while inflammatory cytokines would enable DCs to mount more robust protective adaptive responses against pathogens.

Our present understandings about the Tfh cell differentiation program is that it is initiated by DCs, but for the Tfh cells' final maturation the cognate interaction with B cells is an indispensable step (6, 45). Interaction with B cells is needed for the stabilization of Bcl-6 expression in the Tfh cells (21, 45). The Tfh cells induced by cDC1s express high levels of CXCR5, but they are low on Bcl-6, which suggest that they might represent a pre-Tfh population awaiting interaction with antigen-loaded B cells for their final maturation. It is plausible that the cDC1s, unlike LCs, cannot deliver antigen to B cells in order for the B cells to imprint and stabilize the Tfh cell phenotype.

How do cDC1s prevent the LCs from inducing humoral immune responses? One possible explanation could be that the cDC1s activate T cells faster than LCs, and they are roughly 10 times more potent than LCs at inducing T cell proliferation (24). Thus, we speculate that the fast Th cells imprinting and differentiation induced by cDC1s will prevent the LCs to initiate their program. This hypothesis is supported by our findings that show that if LCs are given time to initiate T cell responses the LC-imprinted T cell phenotype remains stable despite the later presence of antigen-presenting cDC1s (unpublished observation). It also remains to be determined whether Th cells regulate B cell responses by influencing the migration and localization of the DCs. Supporting this idea, we found that MHC class II-knockout LCs express higher levels of CCR7 and lower levels of S1PR1 than WT LCs, which suggest that the cognate interaction with CD4^+^ T cells might be an important factor in the regulation of chemokine receptor expression and intra-nodal localization of DCs. It is possible that cDC1s or high antigen-dose loaded LC-induced Tfh cells cannot license the DCs to migrate to the B cell area, and thus prevent the delivery of antigen to B cells.

The antigen-specific B cells showed no signs of activation in the first week post immunization. The Tfh responses peaked at day 4 (transgenic) or day 7 (endogenous) (26). Thus, these findings suggest a sequential activation model of T and B cells (Graphical Abstract). As such, LCs interact first with naïve T cells to promote Tfh cell formation, which in turn could license the LCs to migrate to the T/B border and B cell area. Here, LCs could present antigen to B cells, probably by forming 3-cell conjugates with both Tfh and B cells. This model is also supported by the fact that LCs still contain intact antigen (both intracellular and surface bound) 7 days post-immunization that could be presented to B cells. Antigen presentation to B cells by DCs has been documented in multiple instances. DCs can present intracellular antigens to make them available to B cells (13), and DCs entering B cell follicles have also been described (19). Whether DCs entering B cell follicles, in addition to presenting antigen to B cells, can also contribute to isotype switching and affinity maturation remains to be determined. In chickens, it has been shown that a DC-like population localized around the splenic ellipsoids can pick up antigens, migrate to the B cells zone, initiate GCs and differentiative into follicular dendritic cells (FDCs) (60). Hypothetically, DCs could have functions that are similar to FDCs and could also facilitate the encounter of antigen-specific T and B cells. *In vitro* studies provided evidence that direct interaction of DCs with B cells is needed for isotype switching (12). Also, DC-derived cytokines, such as IL-12 and IL-6/IL6R were implicated in driving IgM plasma cell or memory B cell responses, respectively (7, 17, 18). IL-6 was first described as a B cell-helping cytokine, thus it is tempting to hypothesize that DC-derived IL-6 not only affects Th polarization but might also have a direct effect on B cells as well. The mechanistic reasons why certain DC subsets and not others migrate into the B cell region remain to be determined (61).

Our carrier antibodies selectively target LCs and cDC1s (not the CD8^+^ LN resident DCs) (26). However, the intraperitoneal delivery of our construct targets LCs and cDC1s found both in the skin and skin-draining LNs (26, 33). The rapid T cell activation we observed suggests that the responses presented in this manuscript might be driven by the DCs localized to the LNs and/or by the DCs transiting to the LNs. Further studies will be needed to dissect the specific contribution of skin resident vs. LN DCs in driving these responses (26).

Both our *in vivo* mouse and *in vitro* human data support the exquisite role of LCs in induction of GC Tfh cells and humoral immune responses. The NHP data further support the translatability of our data, and contrary to original *in vitro* observation (49) provide evidence that targeting LCs is an effective way to induce protective antibody responses in a complete absence of CD8^+^ T cell responses. It is important to emphasize that our findings do not rule out that LCs in the presence of adjuvants might still be able to drive CD8^+^ T cell responses through cross-presentation. Also, it will be important to perform a side-by-side comparison between steady state LCs and cDC2 (62, 63) to determine their relative contribution in regulating Tfh cell formation and humoral immune responses.

We performed our experiments on naïve mice and NHPs, on steady state DCs, and in the absence of any adjuvants. The efficient humoral immune responses mounted against foreign antigens suggest that adjuvant-free vaccines, if carefully designed could be an alternative to the present-day adjuvanted vaccines. Our findings also suggest that the major limitations of some relatively ineffective vaccines currently in use or in development might be that: (1) they are not formulated to specifically target a certain subset of DCs and/or (2) the antigen dose is not tailored to maximize the intrinsic/pre-programmed capabilities of the specific DC subset. This new and substantial departure from the status quo is expected to overcome problems that have hindered our ability to generate effective vaccines.

## Ethics Statement

The Baylor Institutional Care and Use Committee approved all mouse protocols.

## Author Contributions

AB, QS, and BI designed and performed mouse experiments. JK and SC performed the human *in vitro* studies. VC, ND-B, and RLG did the NHP experiments. SZ performed ELISA, ELISpot, and flow cytometry and supervised in house reagent making and quality assurance. GZ and YL analyzed and interpreted data. BI wrote the manuscript. All authors participated in discussions of experimental results and edited the manuscript.

### Conflict of Interest Statement

GZ and SZ are named inventors on BS&W Research Institute patent applications directed to the use of Langerin targeting. The remaining authors declare that the research was conducted in the absence of any commercial or financial relationships that could be construed as a potential conflict of interest.

## Supplementary Material

Supplementary Figure 1 **(A)** Top: Gating strategy for hIgG4-specific B cells. Upstream gates: live/dump. Middle: hIgG4-specific B cell displayed as CD38 by GL7 to define the GC B cells (CD38^−^GL7^+^). **(B)** LC-induced GC responses are not limited to Langerin targeting. Left: Bone marrow chimeras were generated by irradiating CD45.2^+^ hCD40 mice and reconstituting them with WT CD45.1^+^ bone marrow. Eight weeks later the chimerism in the LNs was determined using flow cytometer. Right: As in **(B)**, but the chimeras were immunized with humanized CD40 (hIgG4) antibody that is specific to human CD40 and the percentage of hIgG4-specific germinal center B cells induced by LCs determined by flow cytometer. Data from at least 2 different experiments were pooled. Each dot represents a separate mouse. ^**^*p* < 0.01. **(C)** cDC1s' inability to drive GC responses is not limited to Langerin targeting. Characterization of hDectin-1 BAC transgenic mice. Top: Gating strategy applied for identification of different DC subsets. Middle: hDectin-1 expression by different DC subsets. Littermate WT control (gray) and human Dectin-1 (black line) mice. One representative experiment out of 3 is shown. Bottom: cDC1s were targeted either through Langerin or human Dectin-1 and the percentage of hIgG4-specific germinal center B cells determined using flow cytometer. Data from 2 different experiments were pooled. Each dot represents a separate mouse. ns = not significant.

Supplementary Figure 2 **(A)** IL-10 does not interfere with binding of anti-Langerin. Detection of 4C7 in LCs 3 days after immunization with 4C7 only (orange line) or 4C7-IL-10 (black line), gray: naïve. **(B)** LCs were targeted with α-Langerin antibody in the absence or presence of IL-10. The IL-10 was either directly linked to the antibody (doc:coh-IL-10) or just mixed with the antibody (& coh-IL-10). Fourteen days later the anti-hIgG4 responses were determined by ELISA. Data from multiple experiments were pooled. **(C)** Extrapolated EC_50_ from **(B)**. Each dot represents a separate mouse. **(D)** LCs were targeted with either 1 or 10 μg of antibodies. Fourteen days later the anti-hIgG4 responses in the serum were assessed by ELISA. Data from one representative experiment out of two is shown. Each dot represents a separate mouse. ^*^*p* < 0.05, ^***^*p* < 0.001.

Supplementary Figure 3 **(A)** Gating strategy to characterize the CD4^+^ T cell responses induced by different DC subsets. Mice were transferred with transgenic TEα cells and immunized through the indicated DC subsets with 1 μg of 4C7-Eα. The phenotype of the TEα cells was assessed by flow cytometry 4 days later, at the peak of the response. Representative flow plots. **(B)** Compiled data from multiple mice. Data from one representative experiment out of two is shown. Each dot represents a separate mouse. ^*^*p* < 0.05, ^**^*p* < 0.01, ^****^*p* < 0.0001, ns = not significant. **(C)** LCs and cDC1s differ on transcription factor levels. Steady state LCs and cDC1s from WT mice were stained with the indicated markers. Data from one representative experiment out of two is shown. Each dot represents a separate mouse. Paired t-test, ^*^*p* < 0.05.

Supplementary Figure 4 LCs and cDC1s acquire similar amounts of antigens. Mice were immunized with 1 μg of 4C7-Eα. LNs were harvested at the indicated timepoints and the hIgG4 levels were determined using anti-hIgG and flow cytometry. Each dot represents a separate mouse. ^*^*p* < 0.05, ^**^*p* < 0.01, ^***^*p* < 0.001, ns = not significant.

Supplementary Figure 5 cDC1s express higher levels of LFA-1 than LCs. LN cell suspension. Upstream gate: live/MHC-II/CD11c/Langerin and then LCs defined as CD11b^+^ CD103^−^ and the cDC1s as CD11b^−^ CD103^+^. Gray: isotype; Orange: LCs; Purple: cDC1s. One representative experiment out of three is shown.
