# Supplementary figures and images for: DC Subsets Regulate Humoral Immune Responses by Supporting the Differentiation of Distinct Tfh Cells
Source: Front Immunol. 2019 May 27;10:1134. doi: 10.3389/fimmu.2019.01134 (PMC6545976; doi:10.3389/fimmu.2019.01134)

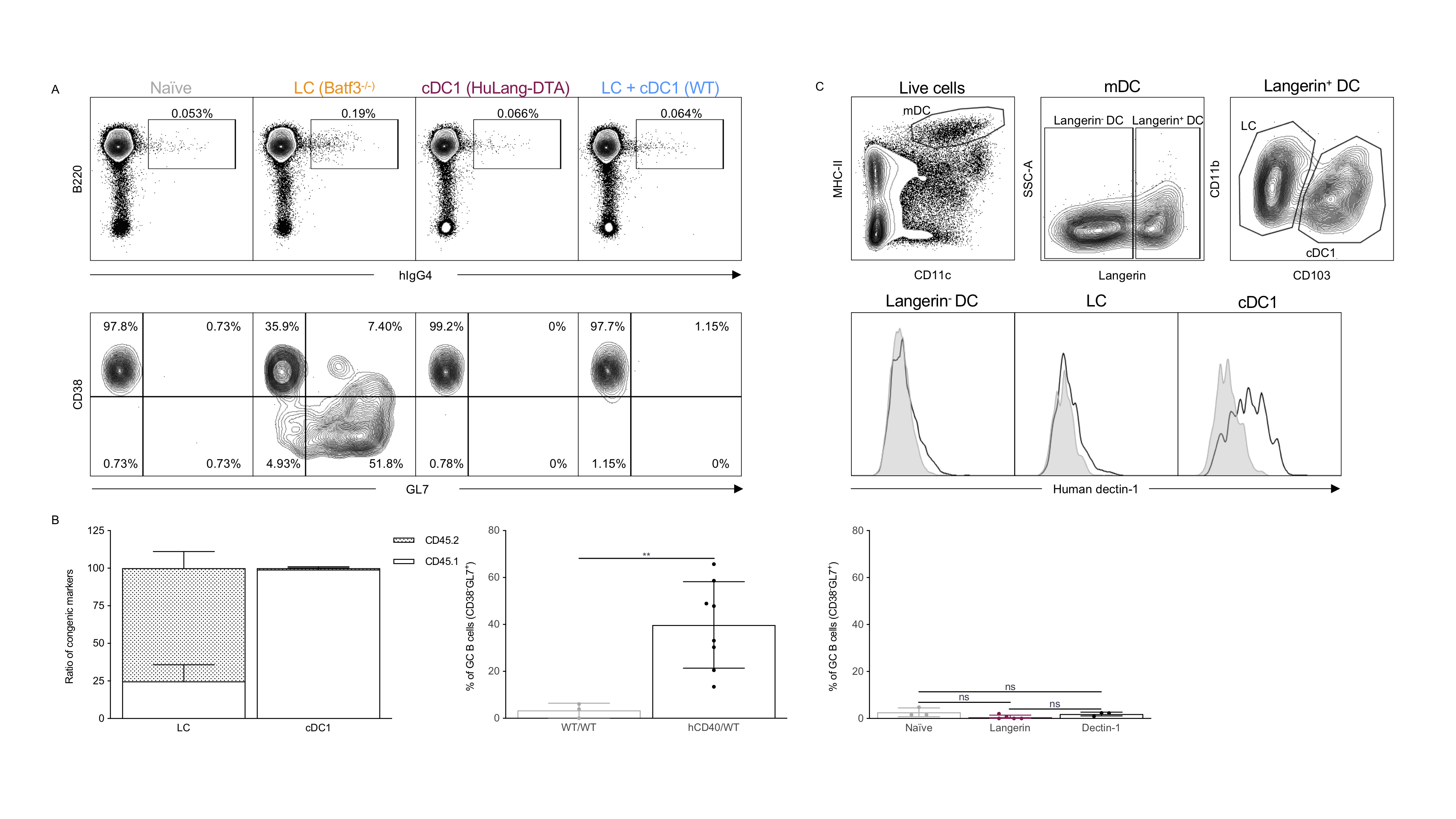

Supplement: Supplementary Figure 1 — (A) Top: Gating strategy for hIgG4-specific B cells. Upstream gates: live/dump. Middle: hIgG4-specific B cell displayed as CD38 by GL7 to define the GC B cells (CD38−GL7+). (B) LC-induced GC responses are not limited to Langerin targeting. Left: Bone marrow chimeras were generated by irradiating CD45.2+ hCD40 mice and reconstituting them with WT CD45.1+ bone marrow. Eight weeks later the chimerism in the LNs was determined using flow cytometer. Right: As in (B), but the chimeras were immunized with humanized CD40 (hIgG4) antibody that is specific to human CD40 and the percentage of hIgG4-specific germinal center B cells induced by LCs determined by flow cytometer. Data from at least 2 different experiments were pooled. Each dot represents a separate mouse. **p < 0.01. (C) cDC1s' inability to drive GC responses is not limited to Langerin targeting. Characterization of hDectin-1 BAC transgenic mice. Top: Gating strategy applied for identification of different DC subsets. Middle: hDectin-1 expression by different DC subsets. Littermate WT control (gray) and human Dectin-1 (black line) mice. One representative experiment out of 3 is shown. Bottom: cDC1s were targeted either through Langerin or human Dectin-1 and the percentage of hIgG4-specific germinal center B cells determined using flow cytometer. Data from 2 different experiments were pooled. Each dot represents a separate mouse. ns = not significant. [file Image_1.jpg]

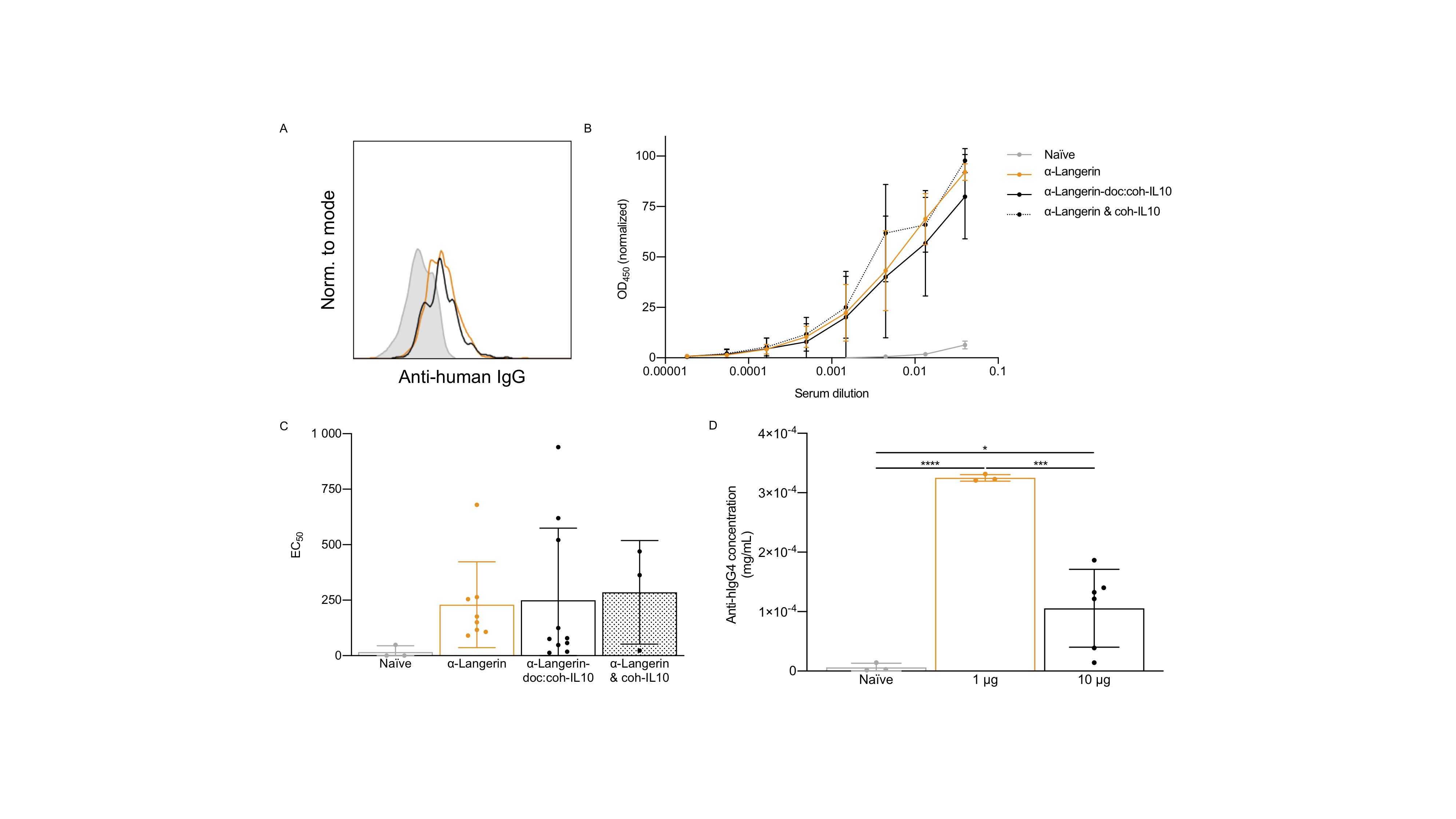

Supplement: Supplementary Figure 2 — (A) IL-10 does not interfere with binding of anti-Langerin. Detection of 4C7 in LCs 3 days after immunization with 4C7 only (orange line) or 4C7-IL-10 (black line), gray: naïve. (B) LCs were targeted with α-Langerin antibody in the absence or presence of IL-10. The IL-10 was either directly linked to the antibody (doc:coh-IL-10) or just mixed with the antibody (& coh-IL-10). Fourteen days later the anti-hIgG4 responses were determined by ELISA. Data from multiple experiments were pooled. (C) Extrapolated EC50 from (B). Each dot represents a separate mouse. (D) LCs were targeted with either 1 or 10 μg of antibodies. Fourteen days later the anti-hIgG4 responses in the serum were assessed by ELISA. Data from one representative experiment out of two is shown. Each dot represents a separate mouse. *p < 0.05, ***p < 0.001. [file Image_2.jpg]

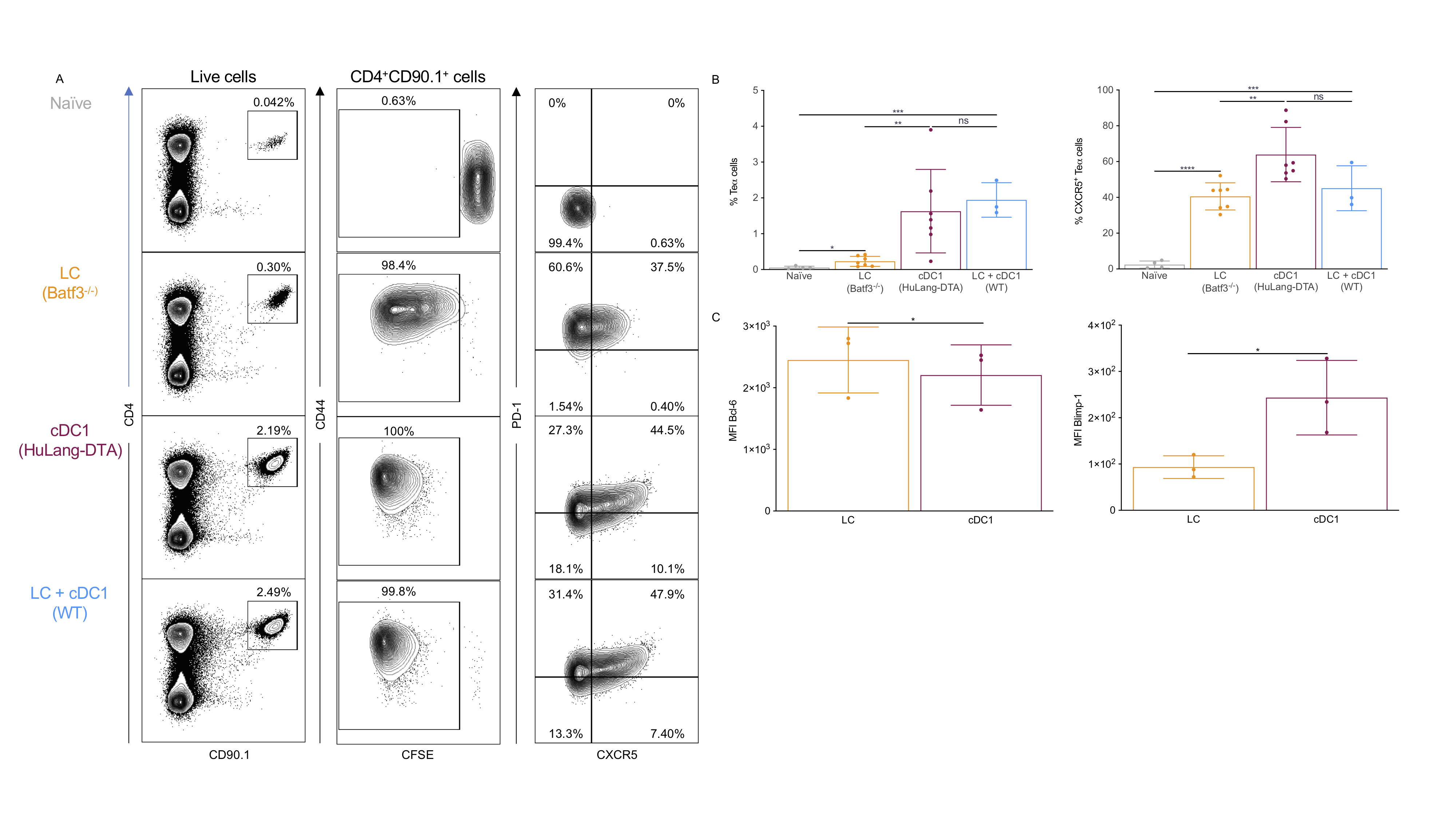

Supplement: Supplementary Figure 3 — (A) Gating strategy to characterize the CD4+ T cell responses induced by different DC subsets. Mice were transferred with transgenic TEα cells and immunized through the indicated DC subsets with 1 μg of 4C7-Eα. The phenotype of the TEα cells was assessed by flow cytometry 4 days later, at the peak of the response. Representative flow plots. (B) Compiled data from multiple mice. Data from one representative experiment out of two is shown. Each dot represents a separate mouse. *p < 0.05, **p < 0.01, ****p < 0.0001, ns = not significant. (C) LCs and cDC1s differ on transcription factor levels. Steady state LCs and cDC1s from WT mice were stained with the indicated markers. Data from one representative experiment out of two is shown. Each dot represents a separate mouse. Paired t-test, *p < 0.05. [file Image_3.jpg]

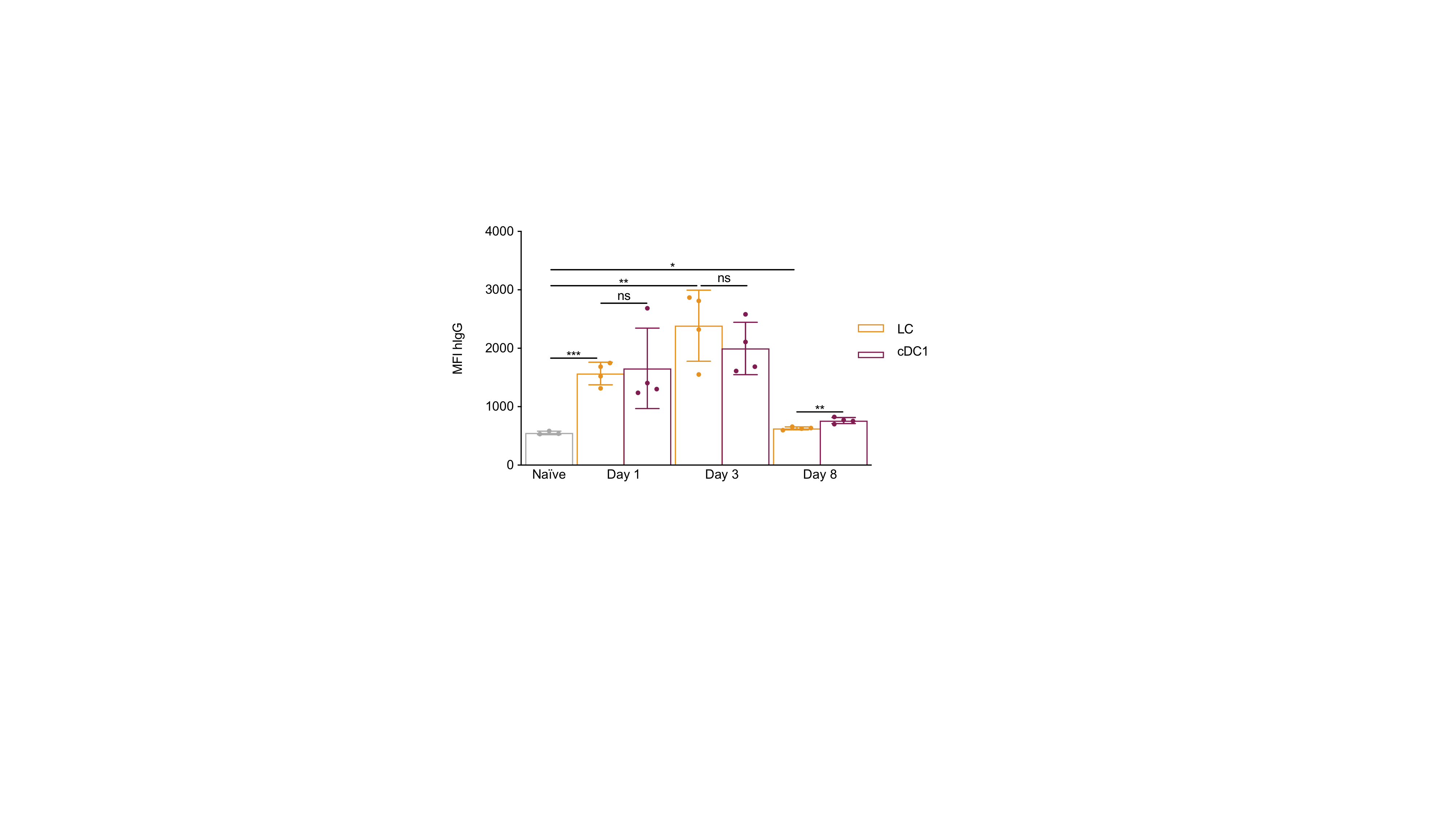

Supplement: Supplementary Figure 4 — LCs and cDC1s acquire similar amounts of antigens. Mice were immunized with 1 μg of 4C7-Eα. LNs were harvested at the indicated timepoints and the hIgG4 levels were determined using anti-hIgG and flow cytometry. Each dot represents a separate mouse. *p < 0.05, **p < 0.01, ***p < 0.001, ns = not significant. [file Image_4.jpg]

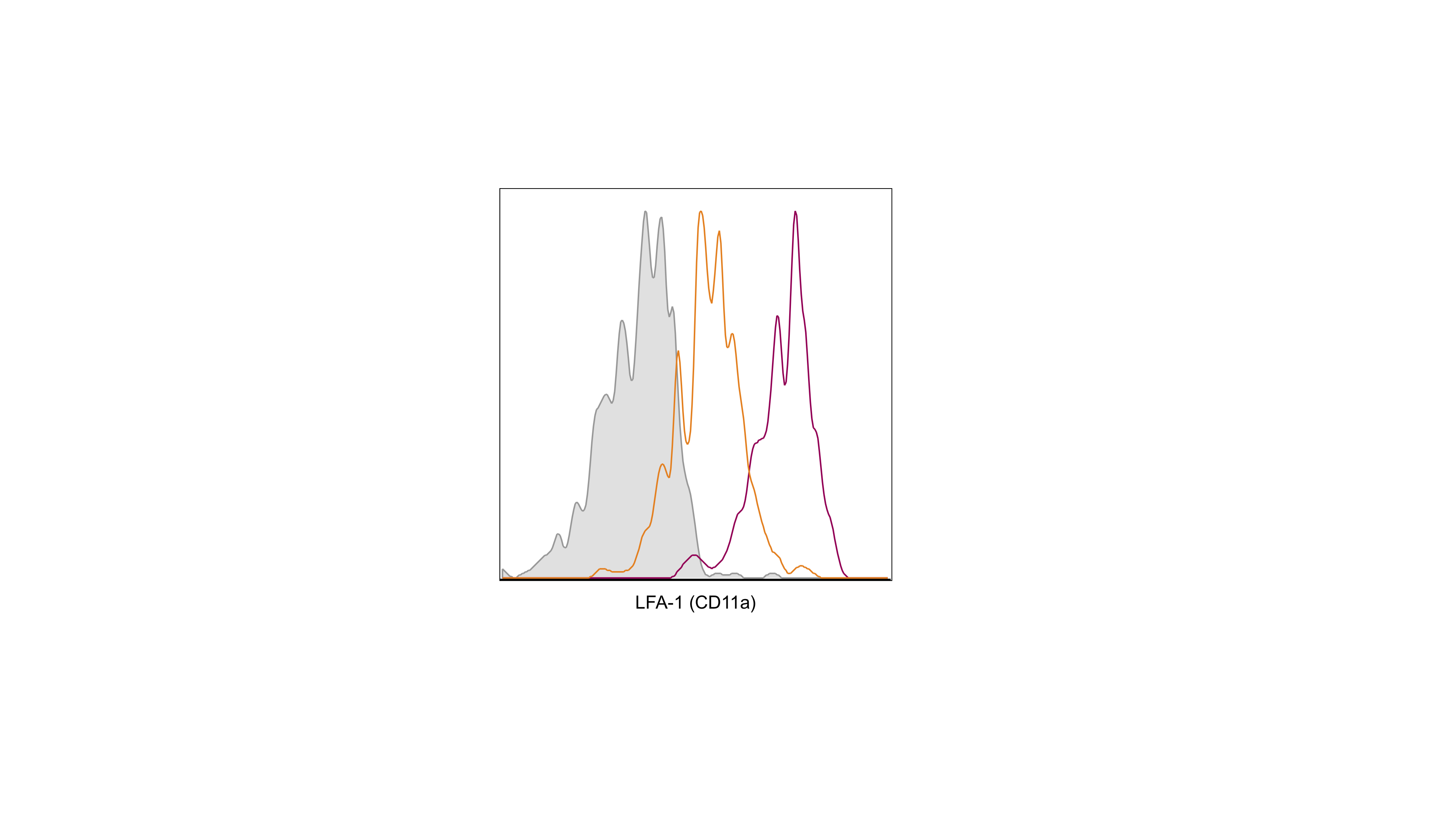

Supplement: Supplementary Figure 5 — cDC1s express higher levels of LFA-1 than LCs. LN cell suspension. Upstream gate: live/MHC-II/CD11c/Langerin and then LCs defined as CD11b+ CD103− and the cDC1s as CD11b− CD103+. Gray: isotype; Orange: LCs; Purple: cDC1s. One representative experiment out of three is shown. [file Image_5.jpg]
